# A Not-So-Gentle Refutation of the Defence of Homeopathy

**DOI:** 10.1007/s11673-015-9682-0

**Published:** 2016-01-05

**Authors:** Jakub Zawiła-Niedźwiecki, Jacek Olender

**Affiliations:** Institute of Philosophy, University of Warsaw, Krakowskie Przedmieście 3, Warszawa, 00-097 Poland; Institute of Philosophy and Sociology, Polish Academy of Sciences, Nowy Świat 72, Warszawa, 00-330 Poland

**Keywords:** Homeopathy, Medical ethics, Evidence-based medicine (EBM)

## Abstract

In a recent paper, Levy, Gadd, Kerridge, and Komesaroff attempt to defend the ethicality of homeopathy by attacking the utilitarian ethical framework as a basis for medical ethics and by introducing a distinction between evidence-based medicine and modern science. This paper demonstrates that their argumentation is not only insufficient to achieve that goal but also incorrect. Utilitarianism is not required to show that homeopathic practice is unethical; indeed, any normative basis of medical ethics will make it unethical, as a defence of homeopathic practice requires the rejection of modern natural sciences, which are an integral part of medical ethics systems. This paper also points out that evidence-based medicine lies at the very core of modern science. Particular arguments made by Levy et al. within the principlist medical ethics normative system are also shown to be wrong.

In their article on the ethics of homeopathy, Levy, Gadd, Kerridge, and Komesaroff (Levy et al. 2015) attempt to defend the indefensible by attacking utilitarian-based medical ethics and by casting doubt on the evidence-based medicine (EBM) paradigm of medical sciences. In our reading, even if their reasoning were sound, it would still be insufficient to defend homeopathy; however, in what follows we also will provide arguments against the reasoning itself. We argue that not only is their critique of utilitarianism wrong but also that any form of a theoretical framework for medical ethics supports the view that homeopathic practice is unethical, unless strong reasons are given for the exceptional status of homeopathy. Moreover, the critique of EBM provided in the article is misleading and mistaken, as it would only be valid if there was an actual distinction between modern science and EBM. We conclude that one must reject modern science or reject the ethicality of homeopathy.

In their critique of utilitarian calculus applied to homeopathy, Levy et al. (2015) try to compare various utilities and disutilities of homeopathy. In this way, they treat homeopathy on a par with evidence-based forms of treatment. This, however, would require a comparison of medical intervention A versus intervention B. Levy et al. (2015) do not do this, nor could they do this. Homeopathy, with its proposed mechanisms of action,^1^ violates the laws of physics and current knowledge in pharmacology, biochemistry, and pathophysiology (House of Commons Science and Technology Committee 2010; Novella 2011; Park 1997). Attempts to prove its efficacy for any condition in clinical science have so far failed (House of Commons Science and Technology Committee 2010; NHMRC 2015). As such, it is not part of established medical practice, and despite it being practised and even funded as medicine, it has the same methodological status as the practice of bloodletting for humoral imbalance in the Middle Ages. Since homeopathy flouts the methodological assumptions of science—and one of the fundamental ethical principles of medicine is to provide the best available care, which in modern medicine means care based on the best available scientific knowledge—in order to ethically justify the use of homeopathy by physicians, one would have to exempt homeopathy from medical ethics.

In order to create the impression that homeopathy is somehow a part of current medical practice, the authors try to distance the concept of EBM both from science in general and from medical practice. According to Levy et al., the standards of EBM need to be relaxed in favour of a “more sophisticated” approach (2015, 207). Indeed, within the philosophy of medicine, there are debates on EBM and its standards of evidence and on ways of translating probabilistic knowledge of populations into actual medical practice that concerns individual patients (e.g., Howick 2011). Another area of discussion is whether a basic plausibility condition should be added to eliminate redundant randomized controlled trials (RCTs) on implausible treatment modalities that are currently practised without an evidence base (Gorski and Novella 2014a, 2014b). Such discussions are concerned with intervention and pathophysiological data that have been arrived at by moving up the EBM’s pyramid of knowledge from basic science through case studies to critical reviews and meta-analyses. However, the issue with homeopathy is not that it is not feasible to organize an RCT or that there are factors that are hard to control for and therefore it is unknown for which members of the population a particular intervention is efficacious. The problem with homeopathy is that it has never been established as efficacious even in laboratory models. Moreover, such proof is highly implausible^2^ due to the fact that homeopathy contradicts the well-established knowledge of the functioning of living organisms and their biochemistry. Still, regardless of its physiological implausibility, homeopathy has been tested in patient populations and never been proven to be efficacious (House of Commons Science and Technology Committee 2010; NHMRC 2015). Therefore, in order for their argument to be valid, Levy et al. (2015) would have to show how homeopathy can be included in medical science. Obviously, they have not done this.

Levy et al. (2015) present a simplified early Popperian approach to scientific evidence: they present the view (by all means logically correct) that *lack of evidence is not evidence of a lack*. It is Popper, with his falsificationism, who presented the view according to which scientific theory can only be disproven (falsified), but it can never be proven in a positive way (Popper 2002; Thornton 2014). However, even Popper defended the ability of science to give positive results thanks to corroboration and probability (Thornton 2014). Popper’s stance was criticized almost immediately, by showing that such an approach treats scientific and pseudoscientific theories equally by making them equally improbable and systematically detached from facts (Lakatos 1978; Kuhn 1996). This is due to the fact that falsificationism only concerns the inner logic of theory (until it is falsified, it is true, at least in part). Although Kuhn (1981) partially agreed with Popper’s idea, he stated that in everyday inquiry scientists need the premises established by the current scientific theory. Lakatos (1981), on the other hand, proposed an approach of falsifying whole groups of theories, or “research programs,” as he called them. Otherwise, the theory would be judged independently from facts (Lakatos 1981). This is because judging each theory independently from all other theories would require suspending any kind of correspondence between them—relating one theory to another in order to confirm or falsify the first one is not a possibility for Popper. Modern approaches to scientific methodology tend to include all possible factors that might affect the outcome of scientific research (Latour 1987). A controversy in scientific inquiry should only be opened if there are serious doubts over issues critical for that particular controversy. Logically, if something contradicts a more basic theory, it automatically contradicts all claims of a higher level. Homeopathy contradicts almost everything we know about physics; therefore, it contradicts all theories based upon it (i.e., biology, chemistry, etc.). The scientific controversy around homeopathy should not be reopened, as no arguments showing a lack of contradiction between homeopathy and the natural sciences have been put forward. The proponents of homeopathy would have to provide an alternative to the current paradigm of the natural sciences, one that would provide explanations at least equally warranted to those current science offers and an explanation of the mechanism of homeopathic remedies. If one wishes to remain within the current paradigm of the natural sciences, one cannot promote homeopathy. On the other hand, if one wishes to support homeopathy and remain scientific, one should provide arguments demonstrating how homeopathy is consistent with science.

Levy et al. demand a “more sophisticated approach to evidence *in* medicine” (2015, 207, *emphasis original*), but they do not offer any kind of methodology outside of EBM that would constitute a basis for the scientific support of homeopathy.^3^ Probably this is because EBM stems from the very core of science and scientific methodology. EBM follows the rule of the best possible evidence (Vos, Willems, and Houtepen 2004) and has even adopted falsificationism in its practice by including the idea of the permanent questionability of each treatment. EBM also acknowledges (in certain aspects) the idea of science being a social construct, subjected to political influence (Djulbegovic, Guyatt, and Ashcroft 2009; Rada, Ratima, and Howden-Chapman 1999). EBM is consistent with the natural sciences and follows the results of basic research, as it does not pretend to create any new scientific theories. It instead applies science to clinical practice and to tests of therapies (Djulbegovic, Guyatt, and Ashcroft 2009). Therefore, if something is placed outside of EBM, it is also placed outside of science (or, rather, the natural sciences). Of course, for example, the sociological research on medical treatments or our philosophical meta-reflections in this paper also might be considered scientific. However, they differ in conditions of acceptance of the hypothesis and cannot be directly compared to examination of medical treatments or to the natural sciences in general. With these arguments, it is clearly evident that homeopathy can be promoted only from outside of science, not by requesting changes within scientific methodology.

If medical science is translated by the theoretical framework of EBM into recommendations for medical practice according to a set of values and goals, then the ethical situation of homeopathy should be seen as either (1) a form of practice that contradicts current knowledge and standards, and yet can be seen as part of medicine, or (2) a form of non-medical practice that is nevertheless offered as part of medicine.

If homeopathy is a form of medical practice (a form of practice that should be included in medicine), we can judge it using the same standards we would apply to any other form of medical practice. Common approaches to medical ethics, regardless of their theoretical justification, require that practitioners apply the best available knowledge to achieve the goals of their actions. In this case, we have two options:

1. Homeopathic practitioners do not possess current scientific knowledge that provides the foundations for medicine—it follows that they are unfit to practise as physicians due to their lack of prerequisite knowledge.
2. Homeopathic practitioners do possess current scientific knowledge that provides the foundations for medicine but choose not to follow it—in which case they are in violation of the commonly accepted principles of medical practice.

In both cases, the practice of homeopathy by physicians is unethical because it violates the principle of medical practice according to the best available knowledge, which in today’s medicine is provided by science.

If, on the other hand, homeopathy is a form of non-medical practice, but is offered in a medical setting, then it is based on mass deception. All major ethical systems provide strong arguments against large-scale deception. Both Kantian and utilitarian arguments are in this case quite compelling. Indeed, in cases of gravely ill patients, the use of homeopathic remedies instead of effective treatments would be one of the emblematic examples of harm caused by deception in a medical setting, which could be avoided, if the patients were not deceived into thinking that they were actually receiving a medical intervention.

At the current stage of science, the claims of the supporters of homeopathic practices contradict the ethics of medicine. In view of the ethical principles of medicine, according to which physicians should practise according to the best available knowledge, those who practise homeopathy must violate the ethics of their profession or the general ethical rule against systematic deception.

It should be clear by now that there is no need to assume any specific normative theory for medical ethics to demonstrate that homeopathy is deeply unethical. However, some of the more specific points made by Levy et al. (2015) should be addressed, as their paper mentions some key notions of the principlist framework in which medical ethics is usually analysed. They argue that opposition to homeopathy is paternalistic and against the autonomy of patients who choose homeopathy. This is a misconstruction of autonomy. An autonomous decision, as typically understood in medical ethics, requires: intentionality, understanding, and voluntariness (Faden and Beauchamp 1986; Beauchamp and Childress 2012). It is not the aim of this critique to present these three issues at length. It is sufficient to say that the condition of understanding requires disclosure that includes the provision of information on efficacy and risk. In the case of homeopathy, such information would have to include information about the lack of scientific evidence of therapeutic efficacy and the lack of rigorous studies concerning the risk of homeopathy as compared to therapies based on biomedical science. One could speak of a patient’s autonomous choice only if, having been provided with this kind of information, he or she still opted for homeopathy. This, however, would not change the fact that a practitioner who practised homeopathy would nevertheless be violating the ethical principle of treatment according to the best available knowledge.

Similarly, principles of beneficence and non-maleficence, regardless of their theoretical justification, are violated (as illustrated above) by either incompetence or deception. The requirement of justice is often violated by these practices on multiple levels as well. The most blatant example is the funding of homeopathy within a public healthcare system whose limited resources could be used to provide effective treatments for the sick. Similarly, in the private medical sector, the use of homeopathy means diverting individual resources from potentially life-saving treatments to methods without proven efficacy. In both cases, resources are spent on practices without proven efficacy, and time is wasted that could be used on therapies with known efficacy and risks. According to World Health Organization (2009) estimates, in the United Kingdom alone this amounted to US$62 million spent on homeopathy in 2007. In the United States, the same report (WHO 2009) estimated the expenditure at US$2.9 billion, whilst healthcare financing has been in increasingly grave crisis worldwide.

In conclusion, the only possible defence of the ethicality of homeopathic practices is a wholesale rejection of modern science. Demands to relax the standards of evaluation of evidence in order to incorporate the pre-scientific theory of Hahnemann (1849) into medicine are tantamount to demands for special treatment. Levy et al. (2015) did not give reasons for such special treatment, nor could they do so. Science, logic, and ethics require that homeopathy be removed from the practice of medicine and relegated to the history of medicine.

## Footnotes

1 Homeopathic theory was laid out by Hahnemann (1849). See also the British Homeopathic Association (n.d.). The House of Commons Science and Technology Committee (2010) and the National Health and Medical Research Council (2015) also provide introductions to homeopathic concepts.

2 We do not claim that there is absolute logical impossibility of homeopathy working, but such a situation would require a revolutionary change in a prevailing majority of areas of science, a whole new scientific paradigm. The evidence requiring such change is simply not there, and we find it unlikely for it to be found. We would like to thank an anonymous reviewer for pointing this out.

3 We reject the possibility of proof outside of EBM—as it is a paradigm designed so comprehensively that it allows one to pick out potential positive effects coming from incomprehensible mechanisms.
